# Techno‐Economic and Profitability Assessment of Stand‐Alone Photoelectrochemical Hydrogen Generation Technology

**DOI:** 10.1002/gch2.202500293

**Published:** 2025-08-17

**Authors:** Debby Chun‐Ting Yang, David Adner, Marko Turek, Christian Hagendorf, Chun‐Nan Chen

**Affiliations:** ^1^ National Taiwan University of Science and Technology Taipei 106335 Taiwan; ^2^ Fraunhofer Center for Silicon‐Photovoltaics CSP 06120 Halle (Saale) Germany; ^3^ The Martin Luther University of Halle‐Wittenberg 06108 Halle (Saale) Germany; ^4^ Freiberg Instruments GmbH 09599 Freiberg Germany; ^5^ Hochschule Anhalt 06366 Köthen Germany

**Keywords:** financial feasibility analysis, green hydrogen, photoelectrochemical cell, techno‐economic analysis, water splitting

## Abstract

Hydrogen production from renewable energy sources without CO_2_ emissions forms a fundamental pillar of the emerging hydrogen‐based economy. Hydrogen technologies demonstrate significant potential for energy storage and integration across chemical and materials industries. Direct solar‐to‐hydrogen (STH) conversion via photoelectrochemical (PEC) water splitting is technologically feasible but has not yet been commercialized. A techno‐economic and financial viability assessment is performed on stand‐alone PEC reactors operating in Germany. A detailed cost structure of the photoelectrochemical reactor is carried out. The total cost of the PEC reactor with a 500 cm^2^ active area is ≈€94.19 based on experimental data. The levelized cost of hydrogen for an off‐grid PEC system in Munich is calculated as €83.71/kg, assuming a 5% STH efficiency. The sensitivity analysis highlights hydrogen production and lifetime as key factors, with hydrogen production determined by STH efficiency and solar irradiance. Upscaling scenarios indicate that achieving a target hydrogen cost of €2/kg is feasible by extending the reactor lifetime to 20 years, reaching 20% STH efficiency, reducing initial capital expenditure by 80%, and securing favorable capital structure with a weighted average cost of capital of 10% or lower. The findings highlight how scaling can support the financial feasibility of PEC hydrogen production.

## Graphical abstract

This study evaluates the levelized cost of hydrogen (LCOH) for a stand‐alone photoelectrochemical reactor. A base‐case LCOH of €83.71/kg is estimated based on experimental data. Sensitivity and upscaling analysis indicate that the €2/kg target is achievable by increasing solar‐to‐hydrogen efficiency, reducing capital expenditure, and extending the reactor lifetime.

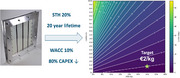

## 1 Introduction

The costs of renewable energy have declined significantly over the last decade, with photovoltaics dropping from 0.42 $/kWh in 2010 to 0.05 $/kWh in 2021.^[^ ^1^ ^]^ This substantial cost reduction has driven an expansion in renewable capacity, with annual photovoltaic installations increasing from 50 GW in 2015 to over 500 GW in 2024. In 2023, renewables contribute 30% of global electricity generation and are expected to grow to 46% by 2030.^[^ ^2^ ^]^ Despite this progress, achieving the goal set by the Paris Agreement (2016) of keeping the global average temperature below 2 °C above pre‐industrial levels requires the development of low‐emissions techniques across all economic sectors, not just electricity. These sectors include industrial production, transportation, heat generation, and agriculture. In 2024, renewables accounted for only 13% of global final energy consumption.^[^ ^2^ ^]^ This indicates that a significant portion of the global energy system must be reconstructed in the following decades.

In many countries and regions, hydrogen is expected to play an important role in the transition to a climate‐neutral economy. Currently, 60 countries have announced concrete hydrogen strategies and roadmaps.^[^ ^3^ ^]^ For example, China aims to produce 1200 ktpa of hydrogen by 2025, and Germany targets 1600 ktpa of electrolysis capacity by 2030.^[^ ^4^, ^5^ ^]^ Although hydrogen is still primarily produced from fossil fuels (≈60% from natural gas and 20% from coal),^[^ ^4^ ^]^ green hydrogen, produced via water electrolysis using renewable electricity, offers a CO_2_‐free alternative. However, a key challenge associated with this approach is the need for extensive expansion of electricity grid infrastructure to connect electrolyzers with renewable electricity sources.

The cost reduction of green hydrogen production has drawn increasing attention as part of efforts to ensure a stable, reliable, and economically accessible electricity supply in line with climate goals.^[^ ^6^ ^]^ According to an International Energy Agency report, the cost of hydrogen production from renewable sources is projected to drop by 30% in 2030, driven by the falling price of renewable energy and the expansion of hydrogen production scale.^[^ ^7^ ^]^ Nevertheless, a lower cost of hydrogen does not ensure the profitability of hydrogen investments, as it also depends on market prices, policy incentives, and subsidy schemes.^[^ ^8^ ^]^

Photoelectrochemical (PEC) hydrogen generation utilizes semiconductor technology to absorb solar energy and directly convert it into chemical energy (hydrogen) within a single device. This approach represents a potential long‐term carbon‐neutral solution with the lowest environmental impact among hydrogen production methods.^[^ ^9^ ^]^ Often referred to as “artificial photosynthesis,” PEC mimics how plants generate fuel from sunlight and water. The systems can be constructed using low‐cost components and do not require rare or precious metals. In addition, they effectively harness solar thermal energy, which is typically considered a waste heat in other hydrogen production approaches. PEC devices consist of relatively few components, which may reduce system complexity and overall production cost; however, deployment at scale requires distributed infrastructure to collect hydrogen across a larger area.^[^ ^10^ ^]^

PEC hydrogen systems have only been studied for a few years. The first large‐area (100 cm^2^) cell was constructed in 2014.^[^ ^11^ ^]^ Nowadays, the most advanced systems achieve a solar‐to‐hydrogen (STH) efficiency of 19%, though many systems only have ≈5% efficiency.^[^ ^12^, ^13^ ^]^ While many cells have only been studied in the laboratory, a photoelectrochemical solar farm with a 100 m^2^ absorber area was constructed in 2021 and operated for 3 months under application conditions.^[^ ^14^ ^]^ Overall, the current technology readiness level (TRL) of PEC is ≈5 (validated in relevant environment) to 7 (system prototype in an operational environment) on the European Commission scale.^[^ ^15^ ^]^

Though there is currently no commercial PEC system on the market, a number of start‐up companies are developing commercial solutions.^[^ ^16^, ^17^ ^]^ The economic prospects of photoelectrochemical water splitting have been examined in several studies. In 2016, Shaner et al. published a techno‐economic analysis of photoelectrochemical and photovoltaic‐electrolytic solar‐hydrogen production systems for a hydrogen output of 10 t/day.^[^ ^18^ ^]^ They estimated a production cost of $300/m^2^ using 2014 as the base year for the photoelectrochemical system, with the membrane (Nafion) accounting for 17% of the cost, the absorber (silicon) for 16%, and the chassis (polypropylene) for 11%. Assuming 10% efficiency and a 20‐year lifetime, they calculated a levelized cost of hydrogen (LCOH) of $11.4/kg and identified system efficiency as the dominant factor. Grimm et al. in 2020 reported an LCOH of $8.43/kg for the same efficiency, based on a module cost of $153.7/m^2^, but highlighted considerable uncertainty in the result.^[^ ^19^ ^]^ Fehr et al. estimated that achieving an LCOH below $2/kg requires 20% STH efficiency, a 10‐year lifetime, and panel costs under $50/m^2^.^[^ ^20^ ^]^ In comparison, Schneidewind et al. projected that reaching $1.5/kg would necessitate >26% STH efficiency and PEC cell costs below $1500/m^2^,^[^ ^21^ ^]^ highlighting how differences in input factors such as efficiency, lifetime, and capital cost can lead to variation in the calculated LCOH.

While economic research on grid‐connected systems is widely available, studies focusing on off‐grid PEC systems remain limited. Due to the limited experimental data, previous techno‐economic analyses largely relied on estimated material and construction costs. For instance, Grimm et al. (2020) drew upon electrolyzer cost data from Shaner et al. (2016) and PEC module component costs from Zweibel (2000) and James et al. (2009), both of which are based on earlier thin‐film data.^[^ ^18^, ^19^, ^22^, ^23^ ^]^ The underlying design approaches may also present economic limitations, as demonstrated by Shaner and Grimm, whose concepts rely heavily on costly membrane components. In contrast, this study incorporates firsthand experimental results and market‐based cost data to provide a more practical assessment of PEC systems, helping bridge the gap between assumed parameters and experimental data. More experimental and public cost data are essential for investors, entrepreneurs, and policymakers to make informed decisions and guide the strategic expansion of hydrogen infrastructure.

This study assesses the financial feasibility of photoelectrochemical reactors by analyzing their cost structure, focusing on material and construction costs based on experimental data collected in this study, and calculating the LCOH. A sensitivity analysis is conducted to determine the impact of parameter value changes on LCOH. Upscaling cases of reaching the target cost of €2/kg are carried out.

## 2 Experimental Section

### 2.1 Off‐Grid Stand‐Alone PEC

**Figure 1:**
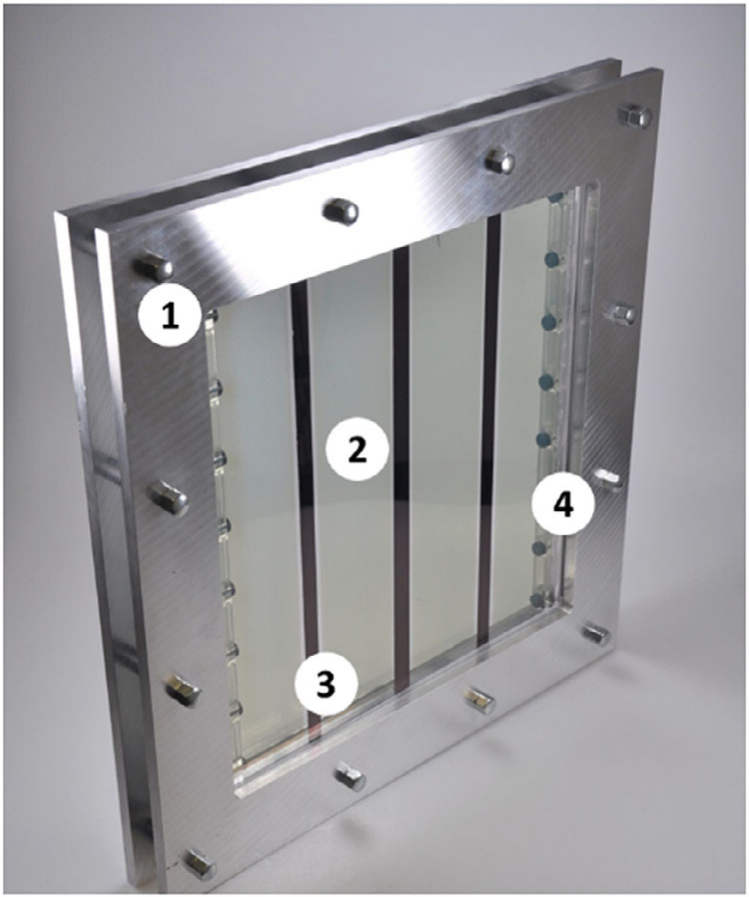
Photograph of the photoelectrochemical reactor (1. closure system with aluminum plate, 2. & 3. cover glass with four sputtered photoelectrodes and three metallic conductors, 4. membrane units with the counter electrode).

Material and assembly costs as part of capital expenditure (CAPEX) were based on data gathered during the construction of a photoelectrochemical module with an absorber area of 500 cm^2^ (**Figure** **1**). It was built from a 300 × 300 × 20 mm^3^ polymethyl methacrylate plate in which a 260 × 260 × 10 mm^3^ cavity was milled for the electrolyte. The absorber was sputtered onto a 300 × 300 mm^2^ glass plate to form four 200 × 50 mm^2^ photoelectrodes separated by three 300 × 10 mm^2^ stripes of metallic conductors for charge extraction. Counter electrodes (Pt wire or IrO_x_/RuO_x_ on Ni wire) were placed on both sides of the reactor. Two proton‐conducting membranes (size 260 × 10 mm^2^), which were mechanically supported by a polystyrene structure, separated both half‐reactions. Hydrogen and oxygen were collected separately on the upper reactor side and exited the reactor through silicon tubes. The reactor body and the glass plate with the absorber were pressed together with a metal frame. Voltage was provided by a custom‐made silicon PV mini module behind the absorber. The system was built based on a CAD model in a professional workshop in Germany.

The hydrogen production rate was calculated from experimental data for a 25 cm^2^ photoelectrode made from Cu (In, Ga)(Se, S). Hydrogen was measured with a gas chromatograph, as described earlier.^[^ ^24^ ^]^ The solar‐to‐hydrogen efficiency η_STH_ was calculated by the production rate of hydrogen ṅ_H2_, the Gibbs free enthalpy of the water splitting reaction Δ_R_G, the solar irradiance P_s_, and the absorber area A_e_. (Equation (1))

(1) $$\eta_{STH}=\frac{{\dot{n}}_{H_{2}}\cdot \Delta_{R}G}{P_{s}\cdot A_{e}}$$

### 2.2 Net Present Value Analysis

Levelized cost is an effective indicator to measure technical‐economic benefits. It is often utilized to evaluate the competitiveness of different power generation technologies.^[^ ^25^ ^]^ The concept of the unit cost of hydrogen production (LCOH) is similar to the levelized cost of electricity generation (LCOE) since hydrogen output is usually measured in energy, similar to electricity calculations.^[^ ^26^ ^]^ The relevant reference factors are shown in **Table** **1**.

**Table 1:** The reference factors of LCOH.

Lifetime	WACC
Tax rate	
**Costs**: Capital expenditure (CAPEX) • Material costs: reactor body, closure system, electrical wires, gas system, electrodes, and PV modules • Assembling costs • Installation costs Operating expenditure (OPEX) • Insurance	**Revenues**: Byproducts Subsidies or selling hydrogen to the market. Hydrogen production • Solar to hydrogen efficiency (STH) • Location • Solar irradiance • Optimal tilted angle • Clear sky or diffuses. • The reliability of the PEC module (degradation rate)

LCOH is the production cost per unit of hydrogen, which is mainly calculated by the net present value (NPV) method and considers all costs and revenues throughout the lifecycle of the system.^[^ ^27^ ^]^ NPV is one of the measurements to assess profitability in capital budgeting by corporations. An investment project will usually be accepted when NPV is above 0, which implies the additional value created by the project. LCOH can be calculated when NPV is 0, as in Equation (2).

(2) $$LCOH=\frac{\sum_{t=0}^{10}\frac{\left(CAPEX_{t}+OPEX_{t}\right)}{{\left(1+WACC\right)}^{t}}}{\sum_{t=0}^{10}\frac{H_{2}Production_{t}}{{\left(1+WACC\right)}^{t}}}$$

where CAPEX_t_ is the annual capital expenditures in the year t, OPEX_t_ is the annual operating expenditures, and WACC is the weighted average cost of capital.

### 2.3 Financial Feasibility Analysis

This study applies an R&D‐only perspective, focusing on modules without additional infrastructure since PEC is yet to become commercially feasible.^[^ ^28^ ^]^ PEC reactor material cost data were obtained from first‐hand information and retail sectors, while other data and assumptions (e.g., WACC, scaling factor) were derived from literature and reports. All data related to the financial feasibility analysis of PEC reactors was based on German market values for the year 2023.

For a system with a standard active area of 1 m^2^, a combination of 20 PEC reactors with an individual absorber area of 500 cm^2^ was considered. As shown in Equation (3), the costs of the PEC system were scaled using an assumed scaling factor of 0.78, the same as the default in the H2A model.^[^ ^29^, ^30^ ^]^ Economies of scale would be realized when an increase in production results in a decrease in the average production cost of each unit. Initial capital expenditures were then determined as scaled PEC and assembling labor costs.^[^ ^18^ ^]^

(3) $$SC=BC\times {\left(\frac{SV}{BV}\right)}^{SF}$$

where SC is the scaled cost, BC is the baseline cost, SV is the scaled value, BV is the baseline value, and SF is the scaling factor.

Derived from experimental findings, the STH efficiency of 5% was determined, diverging from prior studies that suggested efficiencies ranging from 10% to 20%.^[^ ^19^, ^20^ ^]^ An average solar irradiance of 4.11 kWh m^−2^ per day was measured for a 33^о^ optimal tilted angle, located in Munich, Germany (longitude: 11.7^о^, latitude: 48.13^о^) from the Photovoltaic Geographical Information System (PVGIS).^[^ ^31^, ^32^ ^]^

The weighted average cost of capital (WACC) was set at 10%, representing green hydrogen as a higher‐risk project to investors.^[^ ^6^, ^23^, ^33^ ^]^ The lifetime of the system was conservatively set for 10 years, contrasting with the more optimistic 20 years assumed in many previous studies,^[^ ^18^, ^19^, ^21^, ^33^ ^]^ primarily due to the lack of reliability data. Operating and maintenance costs, including maintenance and insurance costs, are 2% of the initial capital expenditure.^[^ ^33^ ^]^

The calculations do not consider BEG 31 subsidies, water purification, or the degradation of hydrogen production. Similar to relative studies,^[^ ^18^, ^19^ ^]^ operational downtime was excluded from the assumptions due to limited long‐term degradation data and the current technological readiness level. The residual value of PEC systems was assumed to equal disposal costs after 10 years. Selected technical and financial parameters are summarized in **Table** **2**.

**Table 2:** Summary of technical and financial parameters, referring to the year 2023.

Parameter	Value	References
PEC reactor costs (€)	94.19	
PEC reactor active area (cm^2^)	500	
Scaling factor	0.78	[29, 30]
Lifetime (year)	10	
WACC (%)	10	[6, 23, 33]
Average solar irradiance (kWh/m^2^/day)	4.11	[31, 32]
STH (%)	5	
Assembling costs (€/m^2^)	10	[18]
Initial capital expenditure (€/m^2^)	984.54	
^Operating and maintenance cost (%)^	2	[33]

## 3 Results

### 3.1 Levelized Cost of Hydrogen Production

**Figure 2:**
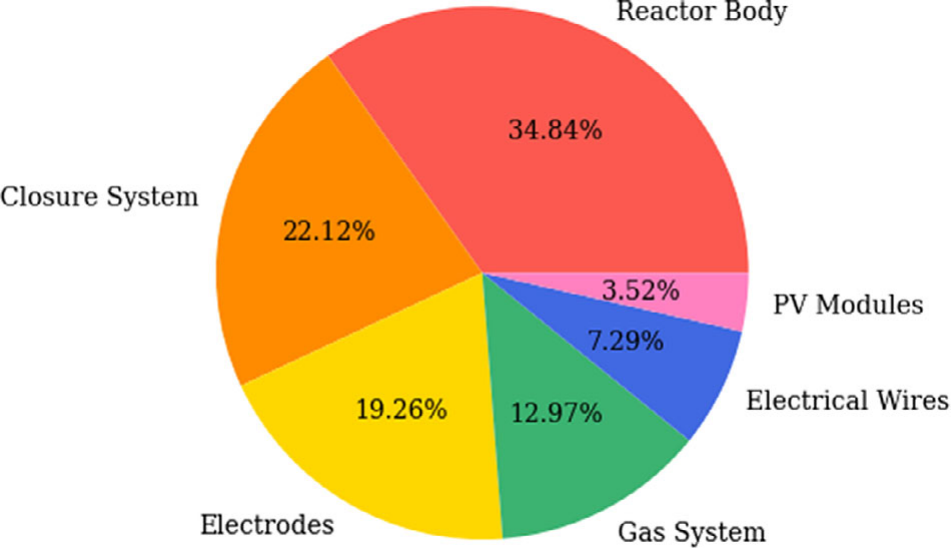
The cost structure of a single PEC reactor with 500 cm^2^ active area. Source: self‐elaboration.

A single PEC reactor with a 500 cm^2^ active area costs ≈€94.19. **Table** **3** presents the costs of a single 500 cm^2^ reactor, broken down into six subsystems: reactor body, closure system, electrical system, gas system, electrodes, and photovoltaic module. The reactor body, the closure system, and the electrodes are the three main costly pieces shown in **Figure** **2**, accounting for 34.84%, 22.12%, and 19.26% of the total costs, respectively. Furthermore, the costs of PEC reactors are primarily impacted by four components: aluminum plate, PMMA plate, cover glass with photoanode, and membrane, comprising 23.42%, 19.48%, 15.97%, and 10.65% of the total costs.

**Table 3:** The main components of six pieces of the PEC reactor.

Pieces	Main Components	Costs (€)
Reactor Body	PMMA plate	29.66
	PS plate	
	Membrane	
	O‐ring	
Closure System	Aluminum plate	27.88
Electrical Wires	Wires	6.21
	Silver varnish	
	Screw‐in sockets	
Gas System	Screw‐in nozzles	11.04
	Silicon hoses	
	T‐hose connectors	
Electrodes	Cover glass with photoanode	16.4
	Platinized titanium wire	
PV	Solar Modules	3

The total cost per module is expected to decrease with an estimated scaling factor of 0.78 as larger quantities are manufactured. Using Equation (1), the initial capital expenditure for 20 units of PEC reactors (including assembling costs) is calculated to be €984.54 m^−2^.

Given the technical and financial parameter assumptions, a 1 m^2^ stand‐alone PEC system located in Munich has a LCOH of €83.71/kg. The pie chart in Figure 2. indicates that CAPEX contributes the most to the total cost, accounting for 89.7%, with a value of €75.1/kg, while OPEX accounts for the remaining 10.3%, €8.6/kg of LCOH.

**Figure 3:**
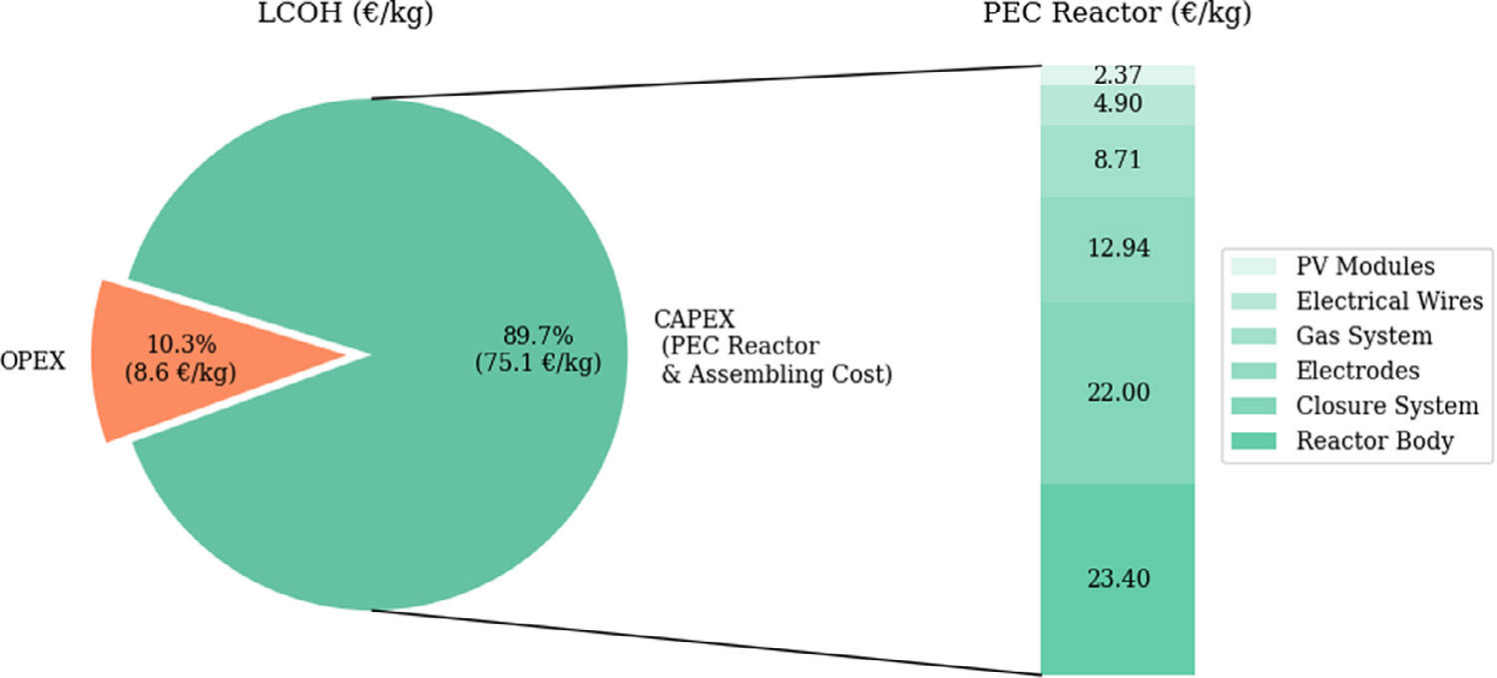
Cost breakdown of the LCOH (€/kg) for the PEC reactor system Source: self‐elaboration.

The bar chart in **Figure** **3** shows the detailed cost breakdown of the PEC reactor. The reactor body, which includes the PMMA plate and membrane, is the most significant influence on CAPEX, contributing 31.49%, equivalent to €23.4/kg. The closure system, including the aluminum plate, is the second impact on CAPEX, accounting for 29.6%, €22/kg. Unlike previous research, the study finds that the main cost contributor is aluminum plate instead of membrane and cover glass with photoanode.^[^ ^28^ ^]^. Furthermore, 12.7% of CAPEX is attributed to the electrodes with PEC‐active glass, with a value of €12.94/kg.

### 3.2 Sensitivity Analysis

**Figure 4:**
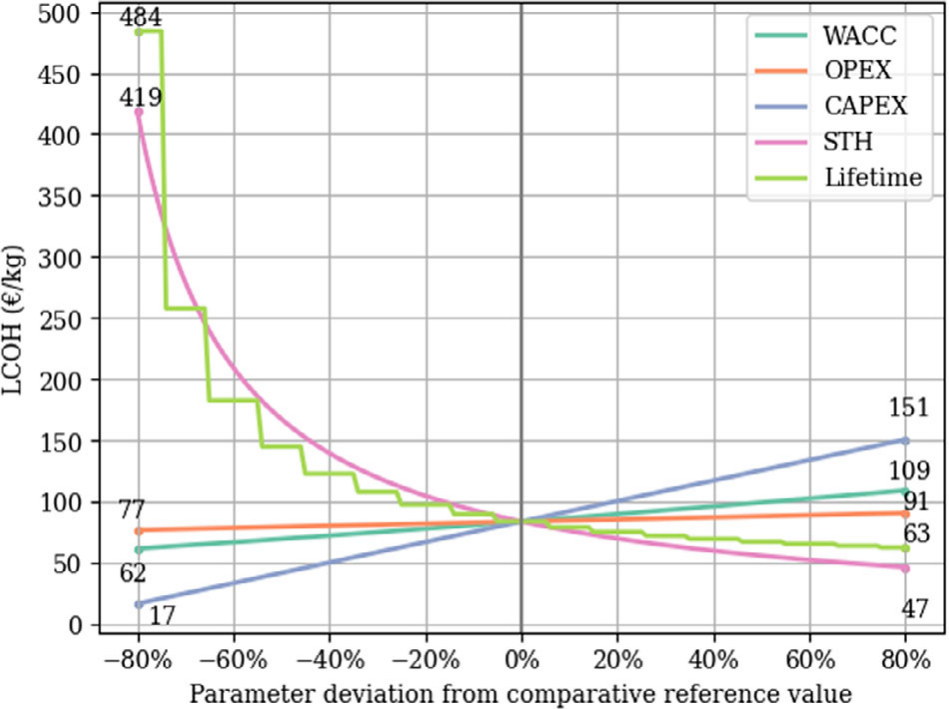
Sensitivity analysis of LCOH for the PEC reactor system in Munich. Source: self‐elboration.

To better understand the impact of different parameters, a comprehensive sensitivity analysis was conducted for the PEC reactor system, with the results shown in **Figure** **4**. The comparative reference value without any parameter changes is €83.71/kg. The study assumed integer values for the operational lifespan, resulting in a decreasing step‐shaped LCOH curve with an increasing lifetime. The lifetime and hydrogen production parameters exhibit a significant and non‐linear effect on LCOH, while other parameters demonstrate a linear impact. A longer system lifetime lowers the LCOH by distributing the capital cost over an extended operating period. Hydrogen production will be affected by factors such as solar irradiance, STH efficiency, and reliability of the PEC reactor. In contrast, the OPEX and WACC lines are less steep, indicating a smaller impact on LCOH from changes.

When all parameters negatively deviate from reference values, lifetime and hydrogen production are the primary parameters affecting LCOH. In such cases, LCOH can be up to five to six times higher than the base case when lifetime and hydrogen production are reduced by 80%. On the contrary, when all parameters deviate positively from their reference values, capital expenditure becomes more critical for LCOH. Capital expenditure higher than the reference case by 80 % would generate LCOH higher by €67.29/kg.

The results underline the greater importance of improvements in technological efficiency (STH) and reductions in initial capital investments (CAPEX) compared to changes in WACC and operating expenditures (OPEX). As WACC reflects not only financing conditions but also project‐related risk, its influence on LCOH is comparatively limited in this analysis. The best‐case scenario for the financial feasibility of PEC reactor systems is the combination of enhanced STH efficiency, optimal solar irradiance, the lowest initial capital expenditure, extended operational longevity, and a low WACC.

To extend the baseline assessment, additional techno‐economic scenarios were simulated to evaluate the impacts of scale and irradiance on hydrogen production costs. At a production scale of 10,000 m^2^, the initial capital expenditure is estimated to decrease to €138.47 m^−^^2^. Under standard Munich irradiance (4.11 kWh/m^2^/day) and 5% STH efficiency, the LCOH is €11.77/kg. Increasing the STH efficiency to 10% lowers the LCOH to €5.89/kg. Under the same efficiency but applying Namibian irradiance (7.4 kWh/m^2^/day), the LCOH further decreases to €3.27/kg.

**Figure 5:**
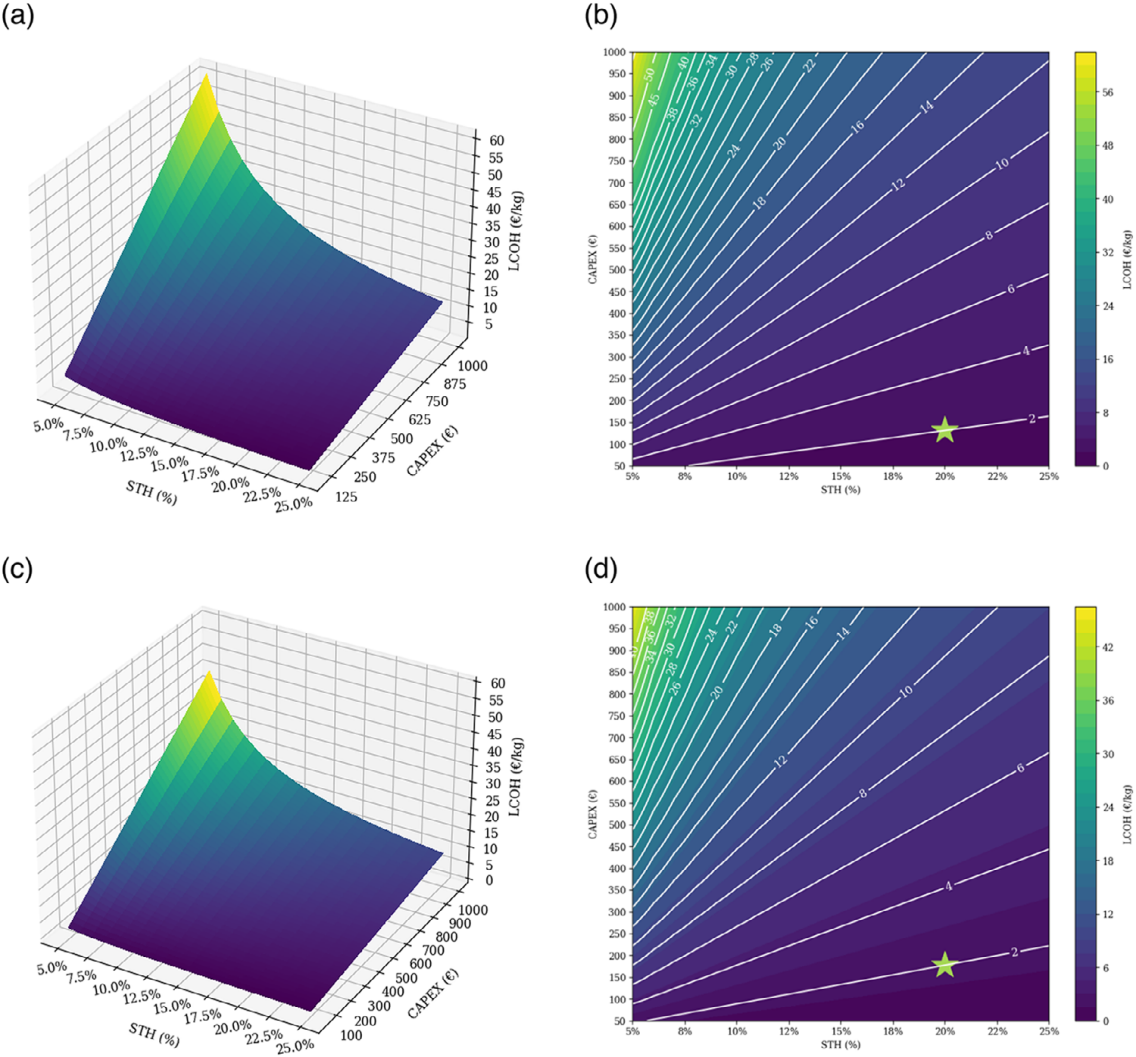
LCOHs of upscaling production under different STH efficiency and CAPEX, assuming a 20‐year lifetime and a project located in Munich. a) 3D plot of LCOHs under 10% WACC. b) Contour plot of LCOHs under 10% WACC. c) 3D plot of LCOHs under 5% WACC. d) Contour plot of LCOHs under 5% WACC. Source: self‐elaboration.

Further analysis of upscaling PEC systems to reach the target cost of €2/kg to €4/kg is presented in **Figure** **5**,^[^ ^34^ ^]^ which illustrates that STH efficiency and CAPEX metrics are used to estimate LCOH under 10% and 5% of WACC, respectively, assuming a 20‐year lifetime, 2% operating and maintenance costs, and a project located in Munich.

3D plots in Figure 5 elaborate on the combined effects of STH efficiency and CAPEX parameters on LCOHs. The gradient, changing blue‐colored into yellow‐colored, represents a lower STH and higher CAPEX, resulting in higher LCOH. As capital expenditure rises, STH efficiency has a more significant effect on LCOH. On the other hand, with higher STH efficiency levels, the LCOH change is relatively lower despite an increase in capital expenditure. Lower WACC, from 10% in Figure 5a to 5% in Figure 5c, has a flattening and compressing effect on LCOH, reducing the variability and value in LCOH, as well as hydrogen production costs, and making investing in PEC systems more attractive.

The graphs allow the extraction of parameters for scenarios in which the €2/kg target cost is reached in upscaling production. The target cost can be achieved with STH efficiency of 20% and CAPEX of €131 under 10% of WACC, as shown in Figure 5b, and with STH efficiency of 20% and CAPEX of €178 when WACC is 5%, as shown in Figure 5d. If the technical progress enhances STH efficiency, producing hydrogen through PEC systems at the cost of €2/kg would be feasible and financially attractive for investors and policymakers.

## 4 Discussion

The sensitivity analysis has shown that the CAPEX investment costs have a major influence on the LCOH. In the design of the reactor, a number of measures were already implemented to reduce manufacturing costs. Sputtering, a cost‐effective and scalable manufacturing process, was used to produce the absorber. The use of materials for components identified as expensive in other studies was minimized through a vertical arrangement. The highest costs were incurred by the reactor body and the closure system. Due to the low production volume, both components were manufactured manually using subtractive manufacturing processes (e.g., milling). When manufacturing larger quantities, processes such as injection molding or injection stretch blow molding can lead to a significant reduction in manufacturing costs.

The efficiency of the modules also has a strong influence on the LCOH. It was assumed to be 5% but must be 20% for competitive hydrogen prices. This efficiency has so far only been achieved on a small area with multilayer absorbers based on III/V semiconductors, but not for complete reactors.^[^ ^12^ ^]^ More research on efficient and cost‐effective absorbers is necessary, but it is also important to develop efficient reactors with minimized losses on the module level, such as electrical resistances, overvoltages, adhering gas bubbles, and unused surfaces. Other options for increasing efficiency could be the use of concentrated sunlight or to make use of waste heat.

The service life of the reactors was assumed to be 10–20 years in this study, primarily due to the lack of reliable long‐term durability data. Hydrogen modules place high demands on the corrosion stability of all materials (e.g., the absorber and the housing materials). Stability tests have already been performed on relevant materials^[^ ^35^ ^]^ and should be continued, extended to whole subsystems (e.g., the entire housing) and to application‐relevant environmental conditions (e.g., light, temperature). Corrosion must also be taken into account in reactor development.

Our study shows that while reducing WACC may improve the bankability of PEC systems, its overall impact on LCOH is limited compared to capital expenditure and system efficiency. What is needed instead is funding for research into absorbers and into the development of reactors, as well as good conditions for start‐ups and entrepreneurs. As soon as a high TRL is reached, targeted policies and subsidies can mitigate high capital requirements and lower LCOH. Measures could include subsidies for large‐area photoelectrode development, tax incentives for scaling PEC,^[^ ^9^, ^36^ ^]^ and demand‐side incentives like consumer rebates and green hydrogen certification. Carbon pricing may enhance competitiveness by internalizing the externalities of fossil fuels. Public‐private partnerships, collaboration with research institutions, and policy advocacy can help drive innovation and integrate hydrogen technologies into energy strategies for large‐scale adoption.

These results suggest that large‐scale deployment can significantly reduce hydrogen production costs, even under modest assumptions regarding efficiency and irradiance. The reduction from €83.71/kg (baseline) to €11.77/kg (10,000 m^2^) highlights the strong influence of scale in lowering capital costs. In addition to direct cost dilution, such scaling may also enable learning curve effects, as observed in photovoltaics, where cumulative production has led to cost reductions through improved manufacturing processes and supply chain maturity. Although further improvements are achievable through higher efficiency and solar irradiance, the marginal cost reductions are smaller in comparison. These findings suggest that efforts to commercialize PEC systems should place early emphasis on scalable, modular designs and manufacturing strategies. However, as the analysis did not isolate scale effects from all other techno‐economic variables, future studies may benefit from a more comprehensive exploration of these interactions.

At the current stage of technological readiness development, PEC faces several challenges in commercialization. From a financial perspective, these challenges can be addressed from two aspects: the supply side and the demand side. On the supply side, current challenges involve the technology still being at the TRL 5. As a result, further technological progress is needed to improve PEC scalability for large‐scale production and support financial feasibility.^[^ ^9^ ^]^ Standardized methods for measuring efficiency also remain underdeveloped.^[^ ^36^ ^]^ On the demand side, the cost of solar hydrogen remains higher than that of other technologies, which may limit market acceptance.^[^ ^37^ ^]^

## 5 Conclusion

Hydrogen production via photoelectrochemical water splitting is a direct method for converting solar energy into hydrogen without carbon dioxide emissions and is an intriguing future possibility in energy transition. PEC technologies remain off the market despite successful realizations in labs and real environments. To bridge the gap and provide practical insights to stakeholders and policymakers, this study presents a financial feasibility analysis of stand‐alone photoelectrochemical reactors in Germany. At current conditions, the estimated base‐case levelized cost of hydrogen for a single stand‐alone (off‐grid) photoelectrochemical system in Munich is €83.71/kg, deriving from experimental results under assumptions with a lifetime of 10 years and STH efficiency of 5% in this study.

Policymakers and investors are interested in the financial feasibility of PEC systems under different financial and technological conditions. Sensitivity analysis in this study shows that system lifetime, STH efficiency, and initial capital expenditure are the dominant factors affecting LCOH, while WACC and operating expenditure have comparatively smaller effects. Therefore, optimal financial viability for off‐grid PEC systems can be achieved through a combination of higher hydrogen production enabled by favorable solar conditions and improved conversion efficiency, lower initial capital expenditure, longer operational lifetime, and a reduced weighted average cost of capital. These findings emphasize that technological progress remains the primary driver of economic feasibility, as reductions in WACC alone are insufficient without corresponding improvements in efficiency, durability, and system design.

This analysis outlines techno‐economically viable pathways for upscaling commercial PEC systems to reach the target cost of €2/kg. Achieving this benchmark requires a combination of favorable conditions, including a reactor lifetime of 20 years, 20% STH efficiency, an ≈80% reduction in capital expenditure, and a WACC of 10% or lower, contingent on project risk and financing structure. Further scenario modeling shows that even with moderate efficiency and irradiance assumptions, large‐scale deployment can significantly reduce hydrogen production costs. At a scale of 10,000 m^2^, the LCOH decreases to €11.77/kg at 5% STH efficiency and to €5.89/kg at 10% in Germany. Moreover, it further drops to €3.27/kg under 10% STH efficiency in Namibia, showing the additional cost advantages of higher solar irradiance. This emphasizes the importance of upscaling system design and cost‐effective manufacturing, as well as the continued need to improve device performance while creating favorable investment and financing conditions.

## Conflict of Interest

The authors declare no conflict of interest.

## Data Availability

The data that support the findings of this study are available from the corresponding author upon reasonable request.
